# C4 Protein of *Sweet Potato Leaf Curl Virus* Regulates Brassinosteroid Signaling Pathway through Interaction with AtBIN2 and Affects Male Fertility in *Arabidopsis*

**DOI:** 10.3389/fpls.2017.01689

**Published:** 2017-09-27

**Authors:** Huiping Bi, Weijuan Fan, Peng Zhang

**Affiliations:** ^1^National Key Laboratory of Plant Molecular Genetics, CAS Center for Excellence in Molecular Plant Sciences, Institute of Plant Physiology and Ecology, Shanghai Institutes for Biological Sciences, Chinese Academy of Sciences, Shanghai, China; ^2^Key Laboratory of Systems Microbial Biotechnology, Tianjin Institute of Industrial Biotechnology, Chinese Academy of Sciences, Tianjin, China

**Keywords:** *Arabidopsis*, AtBIN2, BR-signaling pathway, C4 protein, interaction, male fertility, sweet potato leaf curl virus

## Abstract

Sweepoviruses have been identified globally and cause substantial yield losses and cultivar decline in sweet potato. This study aimed to investigate the interaction between sweepovirus and plant host by analyzing the function of the viral protein C4 of *Sweet potato leaf curl virus*-Jiangsu (SPLCV-JS), a sweepovirus cloned from diseased sweet potato plants in East China. Ectopic expression of the C4 in *Arabidopsis* altered plant development drastically with phenotypic changes including leaf curling, seedling twisting, deformation of floral tissues and reduction of pollen fertility, and seed number. Using bimolecular fluorescence complementation analysis, this study demonstrated that the SPLCV-JS C4 protein interacted with brassinosteroid-insensitive 2 (AtBIN2) in the plasma membrane of *Nicotiana benthamiana* cells. The C4 AtBIN2 interaction was further confirmed by yeast two-hybrid assays. This interaction led to the re-localization of AtBIN2-interacting proteins AtBES1/AtBZR1 into the nucleus which altered the expression of brassinosteroid (BR)-response genes, resulting in the activation of BR-signaling pathway. The interaction of SPLCV-JS C4 and AtBIN2 also led to the down-regulated expression of key genes involved in anther and pollen development, including *SPROROCYTELESS/NOZZLE, DEFECTIVE IN TAPEL DEVELOPMENT AND FUNCTION 1*, and *ABORTED MICROSPORES*, which caused abnormal tapetal development, followed by defective exine pattern formation of microspores and pollen release. Consequently, male fertility in the *C4* transgenic *Arabidopsis* was reduced. The present study illustrated how the sweepovirus C4 protein functioned in host cells and affected male fertility by interacting with the key components of BR-signaling pathway.

## Introduction

Sweepoviruses, named for the sweet potato begomoviruses, are monopartite (no DNA-B, alpha-satellite, or beta-satellite components associated) and phylogenetically distinct from the Old and New World begomovirus groups (Clark et al., 2012). Recently, increasing strains of whitefly transmitted sweepoviruses, mainly sweet potato leaf curl viruses, have been identified globally (Trenado et al., 2011; Wasswa et al., 2011; Albuquerque et al., 2012; Bi and Zhang, 2012; Clark et al., 2012; Pardina et al., 2012; Liu et al., 2014). Besides sweet potato (*Ipomoea batatas*), sweepoviruses also infect other *Ipomoea* species worldwide, such as *I. nil* or *I. setosa* (Trenado et al., 2011), and have been reported to cause substantial yield losses and cultivar decline regionally in some sweet potato varieties (Clark and Hoy, 2006; Ling et al., 2010; Gibson and Kreuze, 2015). As a vegetatively propagated root crop, virus infections in sweet potato often build up over generations. Besides, sweet potato infected by sweepovirus in combination with other virus species is not uncommon (Cuellar et al., 2014). Thus, the build-up and combined infection of viruses provide opportunities for pseudo-recombination and interaction events, a process beneficial to virus evolution and diversity.

The host–pathogen interaction of geminivirus in plants has been extensively studied over the past 20 years. Many reports have shown that geminiviral proteins participate and affect various biological processes of plant cells, including modifying cell cycle regulation, cell proliferation, interaction with the host cell defense system, plant development by interacting with host development-related proteins and cell-to-cell migration of virus through plasmodesmata (reviewed by Lozano-Durán et al., 2011; Hanley-Bowdoin et al., 2013; Lucioli et al., 2016). Like other monopartite geminiviruses, sweepoviruses have small, circular, single-stranded DNA genomes. The DNA virion-sense strand encodes two proteins (V1/coat protein CP and V2), and the complementary strand encodes four proteins. The replication-associated protein (C1 or Rep) and the replication-enhancer protein (C3 or REn) are required for viral DNA replication, and the transcriptional activator protein (C2 or TrAP) has been implicated in the control of viral gene expression. The C4 protein, the smallest protein whose coding sequence is entirely contained within the Rep coding region in a different open-reading frame, plays multiple functions in virus–plant interactions (Hanley-Bowdoin et al., 2013). The C4 of *Beet severe curly top virus* (BSCTV) can up-regulate the RING finger protein to affect BSCTV infection by regulating the host cell cycle (Lai et al., 2009). In some monopartite geminiviruses, C4 has been shown to be associated with the development of disease symptoms, such as leaf curling and vein swelling (Stanley and Latham, 1992; Krake et al., 1998; Gutierrez, 2002). C4 also functions as a suppressor of geminivirus-induced gene silencing in plants (Vanitharani et al., 2004; Gopal et al., 2007; Dogra et al., 2009). The C4 proteins of *Beet curly top virus* (BCTV) were shown to interact with 7 of the 10 members of the *Arabidopsis thaliana* SHAGGY-like protein kinase (AtSK) family (AtSK11, AtSK12, AtSK13, AtSK21, AtSK22, AtSK23, and AtSK32) (Piroux et al., 2007; Deom and Mills-Lujan, 2015; Mills-Lujan et al., 2015) and inhibit phosphorylation of BRI1-EMS-suppressor 1 (BES1). The interactions between C4 protein and SHAGGY-like protein kinases were also confirmed for monopartite *Tomato leaf curl virus* (ToLCV) (Dogra et al., 2009) and bipartite *Tomato golden mosaic virus* (TGMV) (Piroux et al., 2007).

*A. thaliana* SHAGGY-like protein kinases are homologs of the evolutionarily conserved glycogen synthase kinase 3 (GSK3) family of serine/threonine kinases in animals (Doble and Woodgett, 2003). The AtSK gene family has evolved into 10 members possessing diverse functions, of which 7 members have been implicated in the brassinosteroid (BR) signaling pathway. Some of these AtSKs were reported to have redundant roles in BR signaling. Among all the AtSKs, AtSK21 (also known as AtBIN2) acts as a key negative regulator of BR signaling (Vert and Chory, 2006; Ryu et al., 2007; Youn and Kim, 2015). As a class of essential plant hormones, BRs are implicated in regulating broad aspects of plant growth and development, including vegetative and reproductive development, germination, senescence, and responses to various biotic and abiotic stresses (Yang et al., 2011; Fàbregas and Caño-Delgado, 2014; Youn and Kim, 2015). Recent models suggest that AtBIN2 targets two closely related transcription factors, AtBES1 and AtBZR1, which regulate the expression of BR target genes (reviewed by Wang et al., 2012). In the absence of BRs, AtBIN2 acts as an active kinase to phosphorylate and inactivate AtBES1/AtBZR1, which is retained in the cytosol by 14-3-3 proteins or degraded by 26S proteasome (Vert and Chory, 2006; Gampala et al., 2007). In the presence of BR, the hormone binds to BR receptor kinase (BRI1), which initiates a signaling cascade that results in the activation of BRI1-suppressor 1 (BSU1), a phosphatase that inactivates AtBIN2 by dephosphorylating a conserved phosphotyrosine residue. The inhibition of AtBIN2 allows transcription factors BES1/BZR1 to be dephosphorylated by protein phosphatase 2A (PP2A), which accumulate in the nucleus and recruit proteins such as BIM1 and Myb30 to form diverse transcriptional complexes, which bind to promoter regions of BR-responsive genes and consequently regulate their expression (Yang et al., 2011).

Recently, a sweepovirus, *Sweet potato leaf curl virus-*Jiangsu (SPLCV-JS), was identified from diseased sweet potato in East China. Infectivity analysis showed that SPLCV-JS could cause mild symptoms in both *Nicotiana benthamiana* and sweet potato, and this virus could synergize with heterologous beta-satellite DNA to enhance symptom severity and viral DNA accumulation (Bi and Zhang, 2012, 2014). A few studies to date have focused on molecular mechanisms of sweepoviruses, with the exception of epidemic warning (Simmons et al., 2009), genome identification (Albuquerque et al., 2012), and infectivity assays (Trenado et al., 2011; Bi and Zhang, 2014). This study focused on the function analysis of the C4 protein of SPLCV-JS to further investigate the interaction between sweepovirus and plant hosts. Since geminivirus C4 protein could interact with several members of AtSKs in plants and the phenotype of the SPLCV C4-expressing *Arabidopsis* lines was similar to the mutants of BR-signaling pathway genes (Li et al., 2001), this study hypothesized that SPLCV C4 might regulate BR-signaling pathway by interacting with BR-related proteins. This study showed that C4 interacted with AtBIN2 *in vivo*, resulting in the activation of BR-signaling pathway with phenotypic changes, including the abnormal development of tapetum and pollen grains, leading to reduced male fertility in the C4-expressing *Arabidopsis* plants. It demonstrated how SPLCV-JS C4 protein interacted with plants by activating the BR-signaling pathway.

## Materials and Methods

### SPLCV-JS C4 Protein Sequence Analysis

The amino acid sequence of SPLCV-JS (GenBank accession No. FJ176701) C4 was aligned with selected geminivirus C4 protein sequences from GenBank using the ClustalX program. After multiple sequence alignments, phylogenetic analysis was performed with MEGA software version 4.0 using the neighbor-joining method and the bootstrap option (1000 replicates) (Tamura et al., 2007).

## Plasmid Construction

All primers used for gene cloning and plasmid construction are listed in Supplementary Table S1. The SPLCV-JS *C4* gene was amplified by polymerase chain reaction (PCR) from the SPLCV-JS genome using the primer pair C4-5FPK/C4-3RPP. The PCR product was digested with *Kpn*I and *Pst*I, and ligated into the binary vector pCAMBIA1301 (Cambia, Canberra, Australia), which was digested with the same enzymes to construct pCAMBIA1301-C4. Similarly, the full-length sequence of *AtBIN2* amplified from the cDNA of *Arabidopsis* seedlings with the primer pair AtBIN2-5FPK/AtBIN2-3RPP was cloned into the vector pCAMBIA1301 using *Kpn*I and *Pst*I sites to construct pCAMBIA1301-AtBIN2.

For yeast two-hybrid assays, the *C4* was amplified with the primer pair C4-5FPNde/C4-3RPP2, and cloned into the vector pGBKT7 (Clontech) to form a fusion protein with the GAL4 DNA-binding domain (pGBKT7-C4). The N-terminal C4N55 was amplified with the primer pair C4-5FPNde/C4N55-3RP and cloned into the vector pGBKT7 *via* double digestion with *Nde*I and *Pst*I to form pGBKT7-C4N55. The C-terminal C4C31 was amplified with primers C4C31-5FPNde and C4-3RPP2 and cloned into pGBKT7 *via Nde*I and *Pst*I sites to generate plasmid pGBKT7-C4C31. *AtBIN2* was amplified from the cDNA of *Arabidopsis* seedlings with the primer pair AtBIN2-5FPNde/AtBIN2-3RPE and then ligated into pGADT7 (Clontech) through *Nde*I and *Eco*RI sites to form a fusion protein with the GAL4 activation domain (pGADT7-AtBIN2).

For the bimolecular fluorescence complementation (BiFC) assay, the *C4* was PCR-amplified and digested with *Bam*HI and *Spe*I and ligated into vector 35S-N1-YFPN to generate an in-frame C-terminal fusion to the YFP N-terminal fragment downstream of the *CaMV 35S* promoter (C4-YFPN). *AtBIN2* was PCR-amplified and digested with *Bam*HI and *Eco*RI and ligated into vector 35S-N1-YFPC to generate an in-frame C-terminal fusion to the YFP C-terminal fragment (AtBIN2-YFPC).

For subcellular localization, *AtBES1* and *AtBZR1* were PCR-amplified without the stop codon, digested with *Kpn*I and *Not*I and cloned into an intermediate cloning vector that was tagged with eGFP at the C-terminal. Then, the AtBES1-eGFP and the AtBZR1-eGFP fragments were amplified by AtBES1-5FPK/eGFP-3RPB or AtBZR1-5FPK/eGFP-3RPB, respectively, and digested with *Kpn*I and *Bgl*II to ligate into the *Kpn*I- and *Bam*HI-digested expression vector pCAMBIA1301.

### Generation of Transgenic *Arabidopsis* Lines

*Arabidopsis thaliana* ecotype Columbia (Col0) was transformed following standard protocols (Zhang et al., 2006). T1 seeds were collected and germinated on selection media and then plantlets were transplanted to soil in a growth chamber (22 ± 1°C, 16 h light/8 h dark photoperiod). The transgene copy number was confirmed using the Southern blot analysis (Sambrook et al., 2001).

### DNA Extraction and Southern Blot Analysis

The total genomic DNA was extracted from the transgenic *Arabidopsis* plants according to the method developed by Soni and Murray (1994). For the Southern blot analysis, an aliquot of 10 μg of the genomic DNA from each sample was digested with *Hin*dIII and analyzed using a standard protocol for Southern blot (Sambrook et al., 2001). Digested genomic DNA was hybridized with DIG-labeled probe specific to the SPLCV-JS *C4* gene. Labeling, hybridization, and chemiluminescent detection were performed according to the instructions of the manufacturer (Roche Applied Science, Germany).

### Yeast Two-Hybrid Assay

For SPLCV-JS C4 self-activation assay, yeast strain AH109 (Clontech) was transformed with pGBKT7-C4, pGBKT7-C4C31, pGBKT7-C4N55, and the pGBKT7 control. For the C4 and AtBIN2 interaction assay, plasmids pGBKT7-C4, pGBKT7-C4C31, and pGBKT7-C4N55 were co-transformed with plasmid pGADT7-AtBIN2 into the yeast strain AH109. A yeast colony co-transformed with pGBKT7-53 and pGADT7-T plasmids was used as a positive control in the experiment. A yeast strain co-transformed with pGBKT7-lam and pGADT7-T was used as a negative control. All transformants were grown on a dropout medium lacking tryptophan, leucine, histidine, and adenine but containing 5 mM 3-amino-1,2,4-triazole (3-AT) to inhibit residual HIS3 expression. The expression of *LacZ* reporter gene was initially monitored using X-α-Gal as the β-galactosidase substrate.

### BiFC Assay for SPLCV-JS C4 and AtBIN2 Interaction

*Nicotiana benthamiana* plants were grown in pots at 23°C with a 16 h light/8 h dark photoperiod and 60% humidity. Before agroinfiltration, the solutions of *Agrobacterium tumefaciens* harboring binary C4-YFPN or AtBIN2-YFPC were mixed equally. *N. benthamiana* plants grown to the four to five leaf stages were agroinfiltrated with the bacterial suspension using a syringe. *N. benthamiana* plants agroinfiltrated with *A. tumefaciens* harboring C4-YFPN and YFPC or YFPN and AtBIN-YFPC were used as negative controls. The agroinfiltrated leaves were observed under a laser confocal microscope (Carl Zeiss, Jena, Germany) at 36 h post- infiltration (hpi).

### Subcellular Localization of AtBES1/AtBZR1

*N. benthamiana* leaves were agroinfiltrated with the *A. tumefaciens* harboring the expression vectors AtBES1-eGFP and AtBZR1-eGFP, separately. The suspensions of *A. tumefaciens* harboring the expression vector AtBES1-eGFP/AtBZR1-eGFP and pCAMBIA1301-AtBIN2 were mixed equally and used for agroinfiltration to illustrate the effects of AtBIN2 and C4 on the subcellular localization of AtBES1/AtBZR1. In addition, the suspensions of *A. tumefaciens* harboring the expression vector AtBES1-eGFP/AtBZR1-eGFP, pCAMBIA1301-AtBIN2, and pCAMBIA1301-C4 were mixed equally for the agroinfiltration experiment. All agroinfiltrated leaves were observed under a laser confocal microscope (36 hpi).

### BR Sensitivity Analysis

*Arabidopsis* seeds were surface-sterilized and sown on Murashige and Skoog (MS) agar plates with 30 g/L sucrose. The plates were cold-treated for 2 days at 4°C. For root growth inhibition assays, MS plates were placed in a vertical orientation for 4 days in a growth chamber at 23°C with a 16 h light/8 h dark photoperiod and 60% humidity, and the seedlings were then transferred to MS plates amended with 1 μM epibrassinolide (BL) for 5 days. The root length was measured before and after BL treatment, and the hormone sensitivity was measured as root growth inhibition.

### Quantitative Real-Time Reverse Transcription-PCR Analysis

The total RNA was extracted from *Arabidopsis* seedlings or floral buds containing approximately stages 7–9 anthers using the Tiangen Plant RNAprep Pure Kit (Tiangen, Beijing, China). The first-strand cDNA was synthesized using the Takara PrimeScript First-Strand cDNA Synthesis Kit (TaKaRa, Dalian, China) and then used for real-time quantitative reverse transcription-PCR (qRT-PCR) analysis. Primer sequences for each gene are listed in Supplementary Table S2. PCR reactions were performed in a 20 μL volume containing 2× SYBR Green Master Mix (TOYOBO Co., Ltd.), 50 ng cDNA, 400 nM forward primer, and 400 nM reverse primer in a Bio-Rad CFX96 thermocycler. *Actin* or a U-BOX gene *At5g15400* (Ye et al., 2010) was used as a reference control for seedlings or anthers, respectively. All samples were assayed in triplicate and repeated at least twice. The relative expression levels were calculated using the 2^-ΔΔCt^ method (Livak and Schmittgen, 2001).

### Pollen Viability Assay Using Fluorescein Diacetate Staining and Pollen Germination Rate Analysis

Anthers at the anthesis stage were removed and used to brush pollens into the fluorescein diacetate (FDA) staining buffer on the slide. After staining for 20 min, the slide was observed under an optical microscope in blue light (wavelength = 495 nm), and viable pollen grains emitted fluorescence. FDA staining was performed as described by Li (2011). Nikon NIS-Elements BR 3.0 software was used to calculate the pollen viability.

*Arabidopsis* flowers that were just open were picked and the pollen grains were dusted on the solid pollen germination medium (Li et al., 1999) and allowed to germinate for 3–5 h or overnight at room temperature. The pollen germination was observed under a microscope Nikon TE2000-S, and the germination ratio was calculated using Nikon NIS-Elements BR 3.0 software.

### Semi-Thin Section and Light Microscopy Observation

*Arabidopsis* inflorescences were incubated for at least 12 h in formaldehyde–acetic acid–ethanol (FAA) fixing solution (10% formaldehyde, 5% acetic acid, and 47.5% ethanol in water), dehydrated in a graded ethanol series (2× 50, 60, 70, 85, and 95%, and 3× 100%), embedded in epoxy resin and sectioned. Anther transverse sections were stained with 1% toluidine blue at 42°C for 1–2 h (Sanders et al., 1999), bright-field photographs were taken using a Nikon TE2000-S microscope.

### Scanning and Transmission Electron Microscopy

For scanning electron microscopic (SEM) analysis, wild-type and mutant inflorescences were fixed overnight in FAA, dehydrated in a graded ethanol series as described earlier, and samples were critical-point dried in liquid CO_2_. Individual anthers and pollen from flowers that corresponded to a specific stage of wild-type anther development were mounted on SEM stubs. Mounted samples were coated with palladium–gold and then examined under an Autoscan SEM JMS-6360 LV (JEOL, Tokyo, Japan).

For transmission electron microscopic (TEM) analysis, the buds approximately containing anthers at stage 9 were collected and fixed in 2.5% glutaraldehyde in phosphate buffer (pH 7.2) for 4 h at 4°C. After fixation, the tissue was washed and post-fixed with 1% OsO_4_ overnight at 4°C. The samples were then dehydrated in a graded ethanol series, infiltrated with a graded series of epoxy resin in epoxy propane, and embedded in Epon 812 resin. Ultrathin sections were stained with 1% uranyl acetate, followed by lead citrate solution and viewed under a TEM (HITACHI H-7650; Hitachi, Tokyo, Japan).

### Statistical Analysis

All data were presented as the mean ± standard deviation (SD) of at least three independent experiments. Statistical analysis was performed using the SPSS statistical package, version 15.0 (SPSS, IL, United States). Student’s *t*-test was used for analyzing the significance of the differences between the wild-type and transgenic *Arabidopsis* lines. A value of *P* ≤ 0.05 or *P* ≤ 0.01 was indicated by an asterisk (^∗^) or double asterisks (^∗∗^), respectively.

## Results

### Overexpression of SPLCV-JS C4 Protein Alters *Arabidopsis* Development

The 258-bp long open-reading frame of SPLCV-JS C4 encoded a 9.2-kDa protein consisting of 85 amino acids. The amino acids BLAST analysis showed that C4/AC4 proteins were diverse among different strains/isolates. The most similar ones were chosen to perform phylogenetic analysis. Most branches of the phylogenetic tree were less than 70 bootstrap values and the SPLCV-JS C4 protein was grouped together with other sweepoviruses (e.g., SPLCV, SPLCJV, and SPLCBV) (Supplementary Figure S1).

**FIGURE 1:**
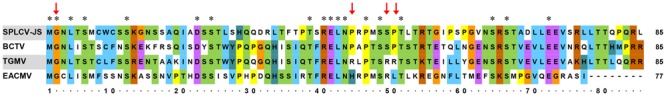
Comparison of amino acid sequences of SPLCV-JS C4 proteins with those of other geminiviruses. Conserved amino acid residues among these sequences are indicated by the asterisks and key amino acid residues related to geminivirus C4 function by red arrows. BCTV, *Beet curly top virus*; TGMV, *Tomato golden mosaic virus*; and EACMCV, *East African cassava mosaic Cameroon virus*.

Further sequence alignment of SPLCV-JS C4 was performed with three well-studied C4 proteins (also known as AC4 in bipartite begomoviruses), including C4 of BCTV, AC4 of TGMV, and AC4 of *East African cassava mosaic Cameroon virus* (EACMCV). The N-terminal G^2^ site in a conserved consensus N-myristoylation motif was required for membrane binding of both the bipartite begomoviruses EACMCV AC4 (Fondong et al., 2007) and the monopartite geminivirus BCTV C4 (Piroux et al., 2007). Besides, the four residues P^44^, A^45^, S^49^, and P^50^ within the central domain of BCTV were reported to contribute to pathogenicity (Piroux et al., 2007). Mills-Lujan et al. (2015) showed that S^49^ was phosphorylated, and this phosphorylation was required for BCTV C4 function. SPLCV C4 showed several conserved amino acid residues, including G^2^, P^44^, S^49^, and P^50^ (**Figure 1**).

**FIGURE 2:**
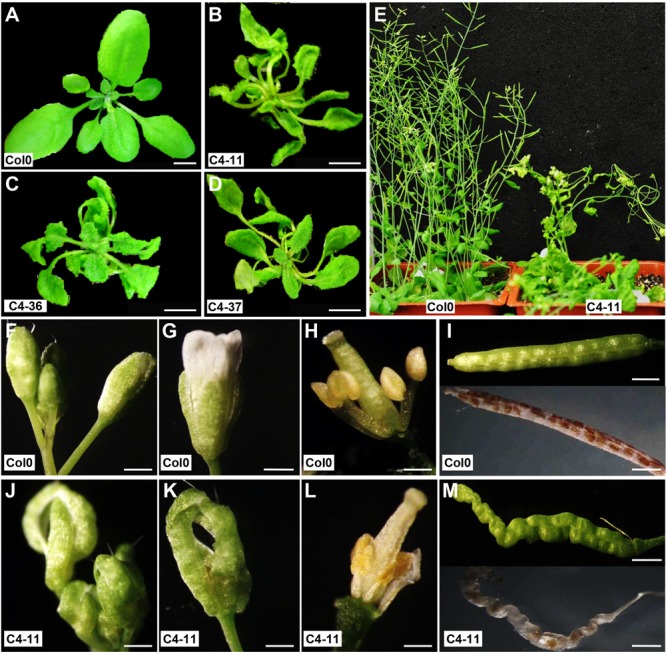
Phenotypic changes of SPLCV-JS *C4* transgenic *Arabidopsis* plant lines. **(A–D)** Seedlings of wild-type Col0 and transgenic lines C4-11, C4-36, and C4-37. **(E)** Flowering *Arabidopsis* plants of wild-type Col0 and C4-11. **(F–M)** Inflorescence, flowers, stamens, pistils, and siliques of wild-type Col0 **(F–I)** and C4-11 **(J–M)**. Scale bar = 1 cm **(A–D)** and 1 mm **(F–M)**.

Transgenic *A. thaliana* lines expressing the C4 protein were produced to better characterize the developmental defects induced by the C4 *in planta* in a system more amenable to genetic studies. The *C4* gene was expressed under the control of the *CaMV 35S* promoter in order to have constitutive expression. After transformation, none of the transgenic *Arabidopsis* T1 generation showed a segregation ratio of 3:1, indicating that the transgene integration was not a single insertion. Indeed, Southern blot analysis showed that all the transgenic lines were multi-copy insertion (Supplementary Figure S2A). Three independent transgenic lines, C4-11, C4-36, and C4-37, were chosen for further study. Real-time qRT-PCR analyses showed that the expression level of *C4* in C4-11 and C4-37 was three or two times higher than that of C4-36 (Supplementary Figure S2B). Compared with the wild type (**Figure 2A**), C4-transgenic seedlings showed elongated and twisted petioles and severely curled leaves (**Figures 2B–D**). During growth, the whole plantlet was distorted and deformed, as illustrated by the C4-11 plant line (**Figure 2E**). Different from Col0 in flowering status (**Figures 2E–I**), the flower buds of the *C4* transgenic plants were not completely enclosed by the sepals and the early flowers were poorly organized (**Figures 2J,K**). The anthers were shrunk (compare **Figure 2H** with **Figure 2L**), and the siliques of the transgenic lines were distorted and had fewer seeds compared with the wild type after destaining (**Figure 2M**). The phenotype severity was in accordance with the C4 expression level in C4-11, C4-36, and C4-37 seedlings (Supplementary Figure S2B), in which C4-11 had the highest C4 expression level with the most severe phenotype.

SPLCV-JS C4 protein was divided into two segments, the N-terminal C4N55 and C-terminal C4C31, to study the functional domains of C4. Both truncated genes were expressed under the control of *CaMV 35S* promoter in *Arabidopsis*. The phenotype of the homozygote *Arabidopsis* is shown in Supplementary Figure S3. No obvious difference was observed between the C4N55 transgenic *Arabidopsis* and the wild-type control. However, the differences between the C4C31 transgenic *Arabidopsis* and the wild-type control were mild, including slightly curved leaves and plants, distorted flowers, and slightly distorted siliques (Supplementary Figure S3). These results demonstrated that the occurrence of severe developmental defects in the transgenic *Arabidopsis* was correlated with the expression of the N-terminal domain of SPLCV-JS C4. Attempts were also made to generate transgenic sweet potato expressing the C4 protein, but no transgenic plants could be produced using the routine protocol (Yang et al., 2011), suggesting that the expression of the SPLCV-JS C4 might be lethal.

### SPLCV-JS C4 Protein Interacts with AtBIN2 to Retain AtBES1/AtBZR1 in the Nucleus and Consequently Up-regulated the BR-Signaling Pathway in *Arabidopsis*

**FIGURE 3:**
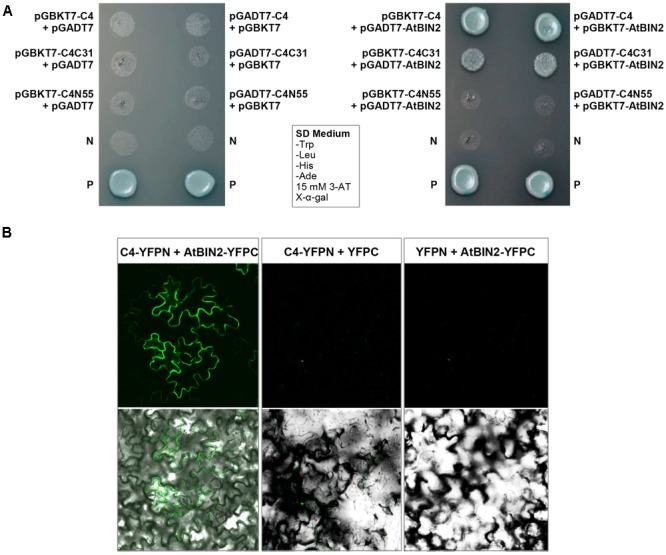
SPLCV-JS C4 and AtBIN2 interaction. **(A)** The interaction of SPLCV-JS C4 with AtBIN2 detected using the yeast two-hybrid assay. Yeast clones grown on dropout medium with X-α-galactosidase (X-α-Gal) and 3-aminotriazole (3-AT, 15 mM) but lacking tryptophan, leucine, histidine, and adenine (SD/-Trp/-leu/-His/-Ade). P, Positive control (pGBKT7-53 and pGADT7-T co-transformant); N, pGBKT7 empty vector. **(B)** Bimolecular fluorescence complementation analysis of the interaction between SPLCV-JS C4 and AtBIN2 in agroinfiltrated *Nicotiana benthamiana* leaf epidermal cells. Leaves were co-infiltrated with C4-YFPN and AtBIN2-YFPC. Leaves co-infiltrated with C4-YFPN and YFPC or YFPN and AtBIN2-YFPC were used as controls. Images were acquired by laser confocal microscopy at 36 hpi.

The interaction between SPLCV-JS C4 protein and AtSK21 (AtBIN2) was confirmed by the yeast two-hybrid assay (**Figure 3A**). The yeast strain co-transformed with the *C4* and *AtBIN2* could grow well in the dropout medium (SD/-Trp/-His/-Ade) supplemented with 15 mM 3-AT and X-α-Gal, and was blue, in both combinations pGBKT7-C4 + pGADT7-AtBIN2 and pGADT7-C4 + pGBKT7-AtBIN2 (**Figure 3A**). When C4 was truncated into two domains, N-terminal C4N55 and C-terminal C4C31, the C4C31 could interact with AtBIN2 weakly, and the C4N55 did not interact with AtBIN2 (**Figure 3A**). Using the BiFC assay, it was further investigated whether the SPLCV-JS C4 protein interacted with the AtBIN2 in plant cells. The C4 alone was localized in both the plasma membrane and the nucleus of *N. benthamiana* protoplasts and epidermal cells as described in a previous study (Bi and Zhang, 2012). AtBIN2 was previously reported both in the cytoplasm and on the plasma membrane (Vert and Chory, 2006). After agroinfiltration of the constructs expressing C4 fused to YFP (C4-YFPN) and AtBIN2 fused with YFP (AtBIN2-YFPC) into intact *N. benthamiana* leaves, a strong fluorescent signal could be detected mainly in the plasma membrane of the co-infiltrated leaves 36 hpi (**Figure 3B**, left panel). No detectable signal was noted in the negative controls (**Figure 3B**, middle and right panels). The result indicated that SPLCV-JS C4 protein interacted with AtBIN2 *in vivo*, and the interaction mainly occurred at the periphery of the cell.

AtBIN2 phosphorylated transcription factors BES1/BZR1 to target them for degradation in the proteasome (Yang et al., 2011). However, AtBIN2 activity is inhibited in the presence of BR. Subsequently, unphosphorylated BES1/BZR1 accumulated in the nucleus and recruited proteins such as BIM1 and Myb30 to form diverse transcriptional complexes to regulate the expression of BR-response genes (Yang et al., 2011). The pattern of AtBES1 and AtBZR1 in the presence or absence of the C4 and/or AtBIN2 was studied to better understand the working mode between SPLCV C4 and AtBIN2 interacting proteins.

**FIGURE 4:**
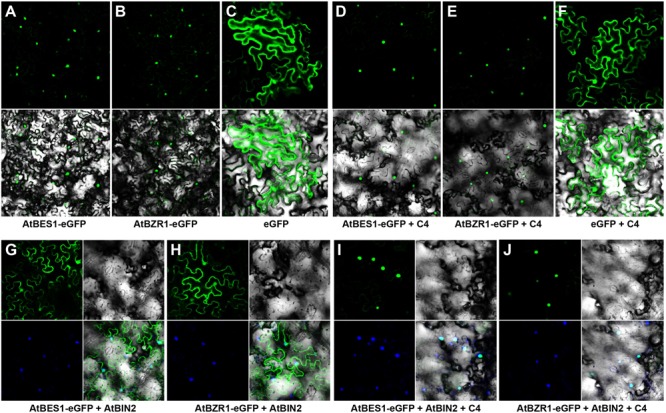
Subcellular localizations of AtBES1/AtBZR1 in *N. benthamiana* epidermal cells in the presence of AtBIN2 and SPLCV-JS C4 proteins. **(A,B)** The nuclear localization of AtBES1 and AtBZR1 using eGFP-fused proteins. **(C)** The eGFP control. **(D–F)** Co-infiltration of AtBES1-eGFP, AtBZR1-eGFP, or eGFP with SPLCV-JS C4. **(G,H)** The changed localization of AtBES1 and AtBZR1 when co-infiltrated with AtBES1-eGFP or AtBZR1-eGFP with AtBIN2. **(I,J)** The re-localization of AtBES1 and AtBZR1 to the nucleus when co-infiltrated with AtBES1-eGFP or AtBZR1-eGFP together with AtBIN2 and SPLCV-JS C4. Images were acquired by laser confocal microscopy 36 hpi. GFP fluorescence, bright-field channel, and DAPI stained merged images are shown, accordingly.

Both AtBES1 and AtBZR1 tagged with eGFP protein were localized in the nucleus in the *N. benthamiana* epidermal cells (**Figures 4A,B**), different from the eGFP control where fluorescence was reported in the cytoplasm and nucleus (**Figure 4C**). When co-infiltrated with C4, the patterns of AtBES1-eGFP and AtBZR1-eGFP in the *N. benthamiana* epidermal cells were unchanged 36 hpi (**Figures 4D,E**) in comparison with the eGFP control (**Figure 4F**), indicating that C4 protein did not directly affect the subcellular localization of AtBES1/AtBZR1. When AtBES1-eGFP or AtBZR1-eGFP were co-infiltrated with AtBIN2, both AtBES1-eGFP and AtBZR1-eGFP were mainly localized at the cell periphery and in the cytoplasm (**Figures 4G,H**), suggesting that the proteins were phosphorylated and relocalized from the nucleus to the cytoplasm. This finding was in accordance with previous reports that AtBIN2 could phosphorylate AtBES1/AtBZR1 and target them for degradation in the proteasome (Gampala et al., 2007; Ryu et al., 2007). In contrast, when AtBES1-eGFP or AtBZR1-eGFP were co-infiltrated with AtBIN2 and SPLCV-JS C4, the fluorescent signal was detected only in the nucleus (**Figures 4I,J**), suggesting that the C4 protein reversed the effects of AtBIN2 on the subcellular localization of AtBES1/AtBZR1 and maintained the accumulation of these two transcription factors in the nucleus.

**FIGURE 5:**
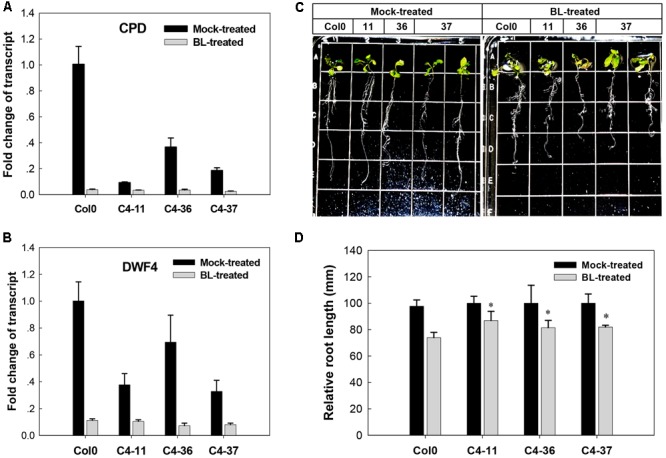
Growth responses of *Arabidopsis* Col0 and SPLCV-JS *C4* transgenic lines to 1 μM epibrassinolide (BL). **(A,B)** Real-time qRT-PCR analysis of the expression level of brassinosteroid biosynthetic genes *CPD* and *DWF4* in Col0 and SPLCV-JS *C4* transgenic lines with or without BL treatment. **(C)** Phenotypic status of Col0 and C4-11, C4-36, and C4-37 plant lines on agar medium 5 days after the BL treatment. **(D)** Root growth repression of Col0 and C4-11, C4-36, and C4-37 plant lines by the BL treatment. Data presented as mean ± SD. Asterisks indicate a statistically significant difference compared with the wild-type value according to the Student’s *t-*test (*P* < 0.05).

The effects of C4 on the phenotype and the expression of two BR biosynthetic genes *CPD* and *DWF4*, which were regulated by AtBES1/AtBZR1 (He et al., 2005; Yang et al., 2011), were further studied in the presence of BL. After treatment with 1 μM BL, the wild-type *Arabidopsis* showed severely curled leaves, elongated and twisted petioles, and shorter taproot with more secondary roots (**Figure 5C**), similar to the C4-expressing lines (**Figures 2B–D**). Real-time qRT-PCR indicated that before BL treatment, the expression of *CPD* and *DWF4* was much lower in the C4-expressing lines than the wild type. After BL treatment, the gene expression of both *CPD* and *DWF4* was repressed to a lower level in all the C4-expressing lines and the wild type, compared with mock-treated samples (**Figures 5A,B**). These data showed that the phenotypes with curled leaves, elongated and twisted petioles, and repressed expression of *CPD* and *DWF4* of C4-expressing lines were similar to those of the wild-type plants treated with BL. These results indicated that, just like the case of BL-treated plants, the BR-signaling pathway was activated in the C4-expressing *Arabidopsis* lines.

### SPLCV-JS C4 Protein Affects Microsporogenesis and Male Fertility in *Arabidopsis*

**FIGURE 6:**
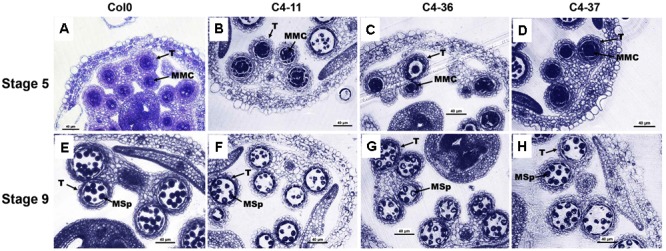
Microspores and anther development in the wild-type Col0 and SPLCV-JS *C4* transgenic plants. **(A–D)** Anthers at stage 5. **(E–H)** Anthers at stage 9. Col0 **(A,E)**; C4-11 **(B,F)**; C4-36 **(C,G)**; C4-37 **(D,H)**. Scale bar = 40 μm. MMC, microspore mother cell; Msp, microspore; and T, tapetum.

The microsporogenesis and pollen morphology of *C4* transgenic lines were studied to further characterize the impact of SPLCV-JS C4 on *Arabidopsis* development. With FDA staining, the pollen viability of C4-11, C4-36, and C4-37 was 15.9, 24.5, and 22.0%, respectively, which was significantly lower than that of wild type (64.9%, Supplementary Figure S4). The pollen germination ratio of C4-11, C4-36, and C4-37 was 26.4, 30.8, and 26.7%, respectively, which was also significantly lower than the 64.4% of wild type (Supplementary Figure S4). The anthers of the C4-expressing *Arabidopsis* lines usually contained fewer pollen grains than that of the wild type. Transverse section of anthers at stage 9 showed that the microspore numbers of C4-11, C4-36, and C4-37 were reduced compared with that of the wild type. Meanwhile, the shape of many microspores of the transgenic lines was abnormal and vacuolated (**Figure 6**).

**FIGURE 7:**
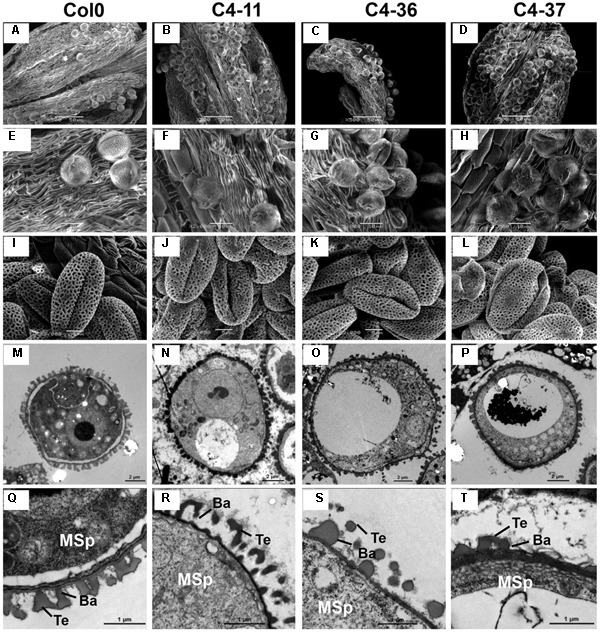
TEM observations of microspores at stage 9 and SEM examination of dehiscent anthers and pollen grains from SPLCV-JS *C4* transgenic lines and the wild type. **(A–H)** Scanning electron micrographs of dehiscent anthers of Col0, C4-11, C4-36, and C4-37. **(I–L)** Scanning electron micrographs of mature pollen grains. **(M–T)** Transmission electron micrographs of transverse section of microspores and the exine ultrastructure of pollen wall at stage 9. Scale bar = 50 μm **(A–D)**, 10 μm **(E–H)**, 5 μm **(I–L)**, 2 μm **(M–P)**, 1 μm **(Q–T)**. Ba, Bacula; Msp, microspore; and Te, tectum.

The anthers at the anthesis stage were also observed by SEM analysis. At this stage, the dehiscent anthers of the wild type contained few pollen grains (**Figure 7A**). A considerable number of pollen grains remained in the inner wall of C4-11, C4-36, and C4-37 anthers (**Figures 7B–D**), indicating that these C4-expressing lines were deficient in pollen release after anther dehiscence, similar to the previously reported BR-related mutants that were defective in pollen release (Ye et al., 2010). Further observation showed that the inner surface of dehisced anthers from C4-11, C4-36, and C4-37 was similar to that of the wild type, but the exines of the pollen grains of the transgenic lines were abnormal (**Figures 7E–H**). Under higher magnification, the wild-type exine had a network-like structure with many lacunae and three narrow apertures (**Figure 7I**). In contrast, the lacunae of C4-11, C4-36, and C4-37 lacked the regularity exhibited by the wild type, and some of the apertures of the transgenic lines were sunken (**Figures 7J–L**). Further examination of pollen development using TEM analysis showed that the microspores of C4-11, C4-36, and C4-37 at stage 9 were poorly organized compared with that of the wild type (**Figure 7M**), and the exine was irregular (**Figures 7N–P**). The complete bacula/tectum structure in the wild type was deficient in the transgenic lines (**Figures 7Q–T**). Moreover, the bacula/tectum structure had almost disappeared in some transgenic plants (**Figures 7R–T**). The abnormal structure of the exine in the *C4* transgenic lines could explain the deficiency of pollen grains release from the inner surface of the anther wall, much like the *bri1-116* mutant of *Arabidopsis* (Ye et al., 2010).

**FIGURE 8:**
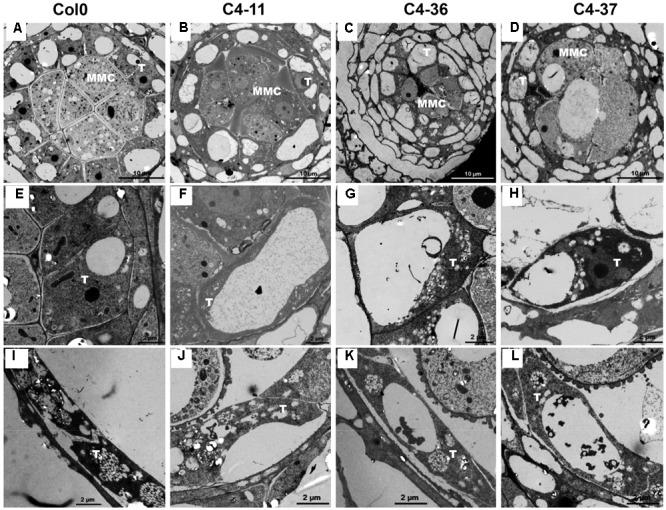
TEM observations of tapetum structure in SPLCV-JS *C4* transgenic lines and the wild type at different developmental stages. **(A–H)** Stage 5, **(E–H)** are enlarged views of **(A–D)**, respectively. **(I–L)** Stage 8. MMC, microspore mother cell; T, tapetum. Scale bars = 10 μm **(A–D)**, 2 μm **(E–L)**.

To investigate the morphological changes that lead to pollen grain defects in the transgenic lines, the anther and microspore morphology of the C4-11, C4-36, and C4-37 lines was examined using TEM. At stage 5, the cell arrangements of tapetum and microspore mother cells of C4-11, C4-36, and C4-37 were irregular compared with those of the wild type (**Figures 8A–D**), and their tapetal cells were larger and more vacuolated also (**Figures 8E–H**). At stage 8, a number of vesicles containing fibrous materials could be found in the tapetum of the wild type (**Figure 8I**), while the tapetum of the transgenic lines was still significantly vacuolated (**Figures 8J–L**). Since tapetum is responsible for the synthesis and secretion of exine material, the abnormal development of tapetum may be an important reason for the reduced male fertility in the *C4* transgenic plants.

**FIGURE 9:**
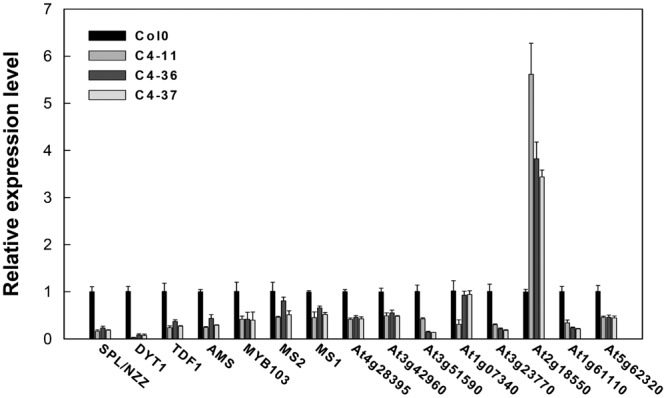
Real-time qRT-PCR analyses of the expression level of several essential genes in anther and pollen development in SPLCV *C4* transgenic lines and the wild-type Col0. The expression level of each gene referred to Col0. The U-BOX gene *At5g15400* was used as an internal reference. Data presented as mean ± SD.

The expression levels of key genes related to the genetic program controlling anther and pollen development in *Arabidopsis* (Ma, 2005) were analyzed in the buds containing anthers at approximately stages 7–9 of the *C4* transgenic lines using qRT-PCR to further identify the effect of C4 on male fertility. The expression levels of *SPROROCYTELESS/NOZZLE* (*SPL/NZZ*), *DYSFUNCTIONAL TAPETUM 1* (*DYT1*), *DEFECTIVE IN TAPEL DEVELOPMENT AND FUNCTION 1* (*TDF1*), *ABORTED MICROSPORES* (*AMS*), *MYB domain protein 103* (*MYB103*), *MALE STERILITY 1* (*MS1*), and *MALE STERILITY 2* (*MS2*) (Ye et al., 2010) were significantly lower than that of the wild type (**Figure 9**). Furthermore, the expressions of several *MS1* downstream genes were measured (Ye et al., 2010). It was found that the expression levels of *At4g28395, At3g42960, At3g51590, At1g07340, At3g23770, At1g61110*, and *At5g62320* in C4-11, C4-36, and C4-37 were lower than that in the wild type, while the expression level of *At2g18550* was higher than the wild type (**Figure 9**). This was in accordance with the case of the BR mutant *cpd* reported previously (Ye et al., 2010). Since AtBES1 could directly bind to the promoter regions of genes *SPL/NZZ, TDF1, AMS, MS1*, and *MS2* to regulate their expression (Ye et al., 2010), C4-activated AtBES1/AtBZR1 modified the expression pattern of downstream genes that were essential for anther and pollen development, consequently affecting the development of tapetum and microspore and leading to reduced male fertility and decreased seed number in the transgenic lines.

## Discussion

Geminivirus C4 proteins are small proteins with diverse functions, and play an important role in virus–plant interaction (Hanley-Bowdoin et al., 2013). The C4 proteins of several geminiviruses have been reported to be symptom-determinant and counteract posttranscriptional gene silencing (Stanley and Latham, 1992; Rigden et al., 1994; Fondong et al., 2007). They are also involved in virus migration (Jupin et al., 1994; Rojas et al., 2001; Teng et al., 2010). Recent studies also found that the C4 protein of BCTV and ToLCV interacts with the plant BR-signaling pathway (Piroux et al., 2007; Dogra et al., 2009; Mills-Lujan and Deom, 2010; Mills-Lujan et al., 2015). The present study demonstrated that the ectopic expression of SPLCV-JS C4 protein in *Arabidopsis* caused severe phenotypic changes and reduced male fertility. The BiFC assay showed that the C4 could interact with AtBIN2 mainly in the plasma membrane to activate AtBES1/AtBZR1 components, so that they could enter the nucleus and activate transcription of BR-response genes.

All transgenic *Arabidopsis* plant lines that showed severe diseased-like phenotypes were the multi-copy insertion of transgene (Supplementary Figure S2A), indicating that single-copy insertion of SPLCV-JS C4 in *Arabidopsis* might be lethal. Transgenic *Arabidopsis* was reported to be produced only when the expression of the BCTV C4 gene was driven by an inducible promoter (Mills-Lujan and Deom, 2010). Transgenic sweet potato plants could not be produced using constitutive expression of SPLCV-JS *C4*, indicating that the C4 protein might be toxic to the embryogenic callus or during somatic embryogenesis.

**FIGURE 10:**
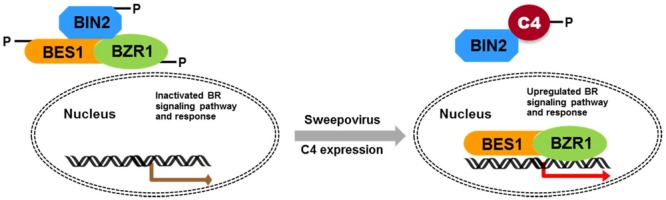
Schematic illustration of SPLCV-JS C4 function in activating BR-signaling pathway. In *Arabidopsis* expressing the sweepovirus C4, AtBIN2 phosphorylated SPLCV-JS C4 to suppress the phosphorylation of AtBES1/AtBZR1, and the unphosphorylated AtBES1/AtBZR1 was released and accumulated in the nucleus to regulate downstream genes expression.

Geminivirus C4 interacts with SHAGGY-like kinases (Piroux et al., 2007; Dogra et al., 2009; Mills-Lujan et al., 2015). The interaction of SPLCV-JS C4 protein and AtBIN2 in this study enabled the release of AtBES1/AtBZR1 in the nucleus to up-regulate the BR-signaling pathway. AtBIN2 has been reported to phosphorylate BCTV C4 at threonine and serine residues (Piroux et al., 2007), specifically serine 49 (Mills-Lujan et al., 2015), which was present in the SPLCV-JS C4. By analogy, SPLCV-JS C4 might act as a competitive substrate of AtBIN2 to suppress the phosphorylation of AtBES1/AtBZR1, and the unphosphorylated AtBES1/AtBZR1 was released and accumulated in the nucleus to regulate the downstream genes expression (**Figure 10**), which was verified by BR-responsive target genes such as *CPD* and *DWF4* (**Figures 5A,B**). Previous studies have reported that the S^49^ residue of BCTV C4 was critical for its interaction with AtSKs and phenotype induction (Mills-Lujan et al., 2015). Besides S^49^, Piroux et al. (2007) also identified additional amino acids P^44^, A^45^, and P^50^ within the central domain of BCTV contributing to the phenotype. In the SPLCV-JS C4 truncations, the C4N55 containing P^44^, S^49^, and P^50^ did not induce obvious altered phenotypes as those induced by intact C4 (Supplementary Figure S3), and C4N55 could not interact with AtBIN2 in the yeast two-hybrid assay (**Figure 3A**). Instead, the C4C31 could induce slight phenotype changes in the transgenic *Arabidopsis* plants (Supplementary Figure S3) and interact with AtBIN2 weakly in the yeast two-hybrid assay (**Figure 3A**). Further experiments are needed to reveal whether and which sites of SPLCV-JS C4 protein are phosphorylated by AtBIN2. Besides AtBIN2, SPLCV-JS C4 may also interact with other AtSKs as the case of BCTV C4 protein (Deom and Mills-Lujan, 2015; Mills-Lujan et al., 2015).

Brassinosteroids are essential regulators of plant growth and development. Many studies have shown that mutations in BR synthesis and signaling components are deleterious for plant growth and reproduction. Ye et al. (2010) demonstrated reduced pollen number, viability, and efficiency in the male reproductive systems of a series of BR biosynthetic and signaling mutants. The AtBES1 targeted genes: *SPL/NZZ, TDF1, AMS, MS1*, and *MS2* that are essential for anther and microspore development (Ye et al., 2010) indeed were found to be down-regulated in their expression in the SPLCV-JS *C4* transgenic *Arabidopsis* (**Figure 9**). Histological, SEM, and TEM analyses of microspores and pollens in SPLCV-JS *C4* transgenic *Arabidopsis* plants also revealed that the expression of C4 caused abnormal tapetum development and pollen maturation, leading to defective exine pattern formation of microspores and pollen release (**Figures 7, 8**), a scenario similar to the BR-related mutants (Ye et al., 2010). Therefore, reduced male fertility in SPLCV-JS *C4* transgenic *Arabidopsis* plants is likely a result of C4-mediated activation of BR-signaling pathway. Interestingly, the *At2g18550*, which encodes homeobox-leucine zipper protein ATHB-21 that interacts with ABA-responsive element binding factor AREB2/ABF4 (Comelli et al., 2012), was significantly upregulated, indicating the crosstalk between BR and ABA signaling pathways (Zhang et al., 2009).

The retention of AtBZR1 in the nucleus as a consequence of SPLCV-JS C4-AtBIN2 interaction also caused transcriptional regulation of many BR-regulated BZR1 target (BRBT) genes. These BRBT genes are related to a wide range of cellular activities and biological processes, including responses to other hormones and environmental stress (Sun et al., 2010), for example, the auxin response factor genes (McSteen and Zhao, 2008); genes encoding auxin/indole-3-acetic acid (IAAs) protein (Nakamura et al., 2003) involved in auxin signaling pathway; abiotic-responsive genes *CBF2, RD29a*; and biotic-responsive gene *WRKY17* (Sun et al., 2010). The expression of *ARF6, CBF2, RD29a*, and *WRKY17* in *C4* transgenic lines was repressed compared with the wild type, while the expression of *IAA19* increased (Supplementary Figure S5), similar to the BL-induced situations (Sun et al., 2010). These changes may worsen the plant phenotype in the C4-expressing lines. The present data indicate that the SPLCV-JS C4 interfered with the BR-signaling pathway, paving a way of understanding how sweepoviruses interacted with their host plants.

Brassinosteroid has already been found to induce disease resistance in plants (Nakashita et al., 2003). The interaction between geminivirus C4 protein and AtBIN2 might be a host defense reaction to tag the C4 protein for degradation (Piroux et al., 2007). Mills-Lujan et al. (2015) reported that plasma membrane-associated BCTV C4 protein interacted with and co-opted multiple AtSKs to promote its own phosphorylation and activation to subsequently compromise cell cycle control. Therefore, SPLCV-JS C4 protein can facilitate its virus infection through interacting with the BR-signaling pathway, but the interaction can also activate the steroid hormone-mediated disease resistance of host plants. Nevertheless, whether SPLCV-JS C4 interacts with other AtSKs or the BR-signaling pathway of sweet potato can be activated by SPLCV-JS during natural infection remains to be investigated in future studies.

Outbreaks of sweepovirus disease have been reported worldwide and caused substantial yield losses and cultivar decline in sweet potato. Nevertheless, sweepoviruses have not drawn much attention of virologists. Only recently several strains or isolates were identified and their infection assays established (Trenado et al., 2011; Bi and Zhang, 2012, 2014). An increase in the understanding of the virus–plant interaction is essential for developing durable and sustainable strategies for biotechnological control of virus pandemics. Therefore, the present study not only disclosed how the SPLCV-JS C4 protein interacted with plant hormone signaling pathway and subsequently affected plant growth and reproduction, but also confirmed that the C4 protein could be used as a target gene for engineering crops resistant to sweepoviruses.

## Author Contributions

HB performed most of the experiments and drafted the manuscript. WF conducted part of yeast two-hybrid assay. PZ coordinated and designed the study and revised the article.

## Conflict of Interest Statement

The authors declare that the research was conducted in the absence of any commercial or financial relationships that could be construed as a potential conflict of interest.
