# Supplementary material for: C4 Protein of Sweet Potato Leaf Curl Virus Regulates Brassinosteroid Signaling Pathway through Interaction with AtBIN2 and Affects Male Fertility in Arabidopsis
Source: Front Plant Sci. 2017 Sep 27;8:1689. doi: 10.3389/fpls.2017.01689 (PMC5623726; doi:10.3389/fpls.2017.01689)
Supplement: Supplementary file 1 [file Data_Sheet_1.PDF]

Supplementary Material

C4 protein of *Sweet potato leaf curl virus* regulates brassinosteroid signaling pathway through interaction with AtBIN2 and affects male fertility in *Arabidopsis*

Huiping Bi, Weijuan Fan, Peng Zhang*

*** Correspondence:** Corresponding Author: zhangpeng@sibs.ac.cn

Supplementary Table S1 Primers used in plasmid construction.

Primer name	Sequence (5'-3')	Plasmid-gene
C4-5FPK	CTCGGTACCATGGGGAACCTCACCTCCATG	pCAMBIA1301-C4
C4-3RPP	TGCCTGCAGTTAGAGCCTCTGCGGCTGCG	
AtBIN2-5FPK	CTCGGTACCATGGCTGATGATAAGGAGATGCCT	pCAMBIA1301-AtBIN2
AtBIN2-3RPP	TGCCTGCAGTTAAGTTCCAGATTGATTCAAG	
C4-5FPNde	TAGCATATGGGGAACCTCACCTCCATGTG	pGBKT7-C4
C4-3RPP2	TGCCTGCAGGTTAGAGCCTCTGCGGCTGC	
AtBES1-5FPK	AGCGGTACCATGAAAAGATTCTTCTATAATTCC	AtBES1-eGFP
AtBES1-3RPN	ATAGTTTAGCGGCCGCACTATGAGCTTTACCATTTCC	
AtBZR1-5FPK	AGCGGTACC ACCACGAGCCTTCCCATTTCC	AtBZR1-eGFP
AtBZR1-3RPN	ATAGTTTAGCGGCCGCACTATGAGCTTTACCATTTCC	
eGFP-3RPB	GACAGATCTTTACTTGTACAGCTCGTCCATG	eGFP
C4YFPN-5FPBam	ACCGGATCCATGGGGAACCTCACCTCCATG	C4-YFPN
C4YFPN-3RPSpe	GATACTAGTGAGCCTCTGCGGCTGCGTCG	
AtBIN2YFPC-5FPB	AACGGATCCATGGCTGATGATAAGGAGATG	AtBIN2-YFPC
AtBIN2YFPC-3RPEc	TTAGAATTCAGTTCCAGATTGATTCAAG	

The underlined sequences indicate restriction enzyme sites.

Supplementary Table S2 Primers used in real-time qRT-PCR analysis.

Gene	qRT-PCR forward primer	qRT-PCR reverse primer
C4	5’-AATCCTCGTCCGATGTCA-3’	5’-GCTGCGTCGTTAGCAGTC-3’
AtActin	5’-GGTAACATTGTGCTCAGTGGTGG-3’	5’-AACGACCTTAATCTTCATGCTGC-3’
DWF4	5’-AAACAACGGAGCGTCATCCTCA-3’	5’-AGCTCTGAACCAGCACATAGCCTT-3’
CPD	5’-TAATGAGACGCTACGAGTGGCTAAC-3’	5’-GTCTAAATGAACCGCTCTAAACGAT-3’
At5g15400	5’-AGAGTGGGAGCAGAGACCAA-3’	5’-ATGTTGGCAACCCTCTCAAC-3’
SPL	5’-TCGCTAGAGCAGCTTCAGTT-3’	5’-CCTCCATTGGTCCCGTAT-3’
DYT	5’-TTATGAGATTTCTCGGATTCG-3’	5’-TCCTGTGTCTGAACAGAGGC-3’
TDF	5’-CGGTTCCTCAAGTAGTGGG-3’	5’-ATGTATTCGGCTTCGATGTT-3’
AMS	5’-TCGTTGCTGGAAATAACCC-3’	5’-TTTGCATAGAGCCTGTAGCC-3’
MYB103	5’-AGATGGAATGACGATGATGAG-3’	5’-GCTTGTAAATCCCACAAGACA-3’
MS2	5’-GTATCAGATCGCTTCTTCGG-3’	5’-TGCATGGGGATGTTTTGTA-3’
MS1	5’-TGGTGGGTGGTCAAATAGAG-3’	5’-TCATCATTCCTACGTTCCCT-3’
At4g28395	5’-ACCAAGAATCCCGACGTT-3’	5’-AGGCTTCCCTTCCCAATA-3’
At3g42960	5’-AAGGGAGTTTGCTGACCG-3’	5’-GACGATTCTTGGCTTGCA-3’
At3g51590	5’-GCGGGTAAAGAAATCAAGG-3’	5’-AAACTGATGGGGTAGGGAAT-3’
At1g07340	5’-CACCAAGAGTGAGAACAGACC-3’	5’-AACATAACCACGTTGATACCG-3’
At3g23770	5’-CATTGGGGAATTTTGAACC-3’	5’-AACCAGAAAACGATCTTCTACC-3’
At2g18550	5’-GCCAATCATACGACACCG-3’	5’-GTAGAGCAAGTTCTCGTCAGC-3’
At1g61110	5’-GCAAATGGTGTTATGGATACG-3’	5’-GAGTGCCAGTTCATGTTAGGA-3’
At5g62320	5’-AAAATGACGAAGGAGGAGGT-3’	5’-TGACGAAGAAGAACATGATGAC-3’
IAA19	5’-GGCTTGAGATAACGGAGCTG-3’	5’-AACCTTCTCAGCGTCACCAC-3’
CBF2	5’-AGACCATGAGCATCCGTCGTCATA-3’	5’-CGGAATCAACCTGTGCCAAGGAAA-3’
RD29a	5’-CCTGAAGTGATCGATGCACCAG-3’	5’-TGGTGTAATCGGAAGACACGAC-3’
WRKY17	5’-CTGCATCACAAGGCTTAA-3’	5’-GGAGCCGGAGTTGTCACTTT-3’

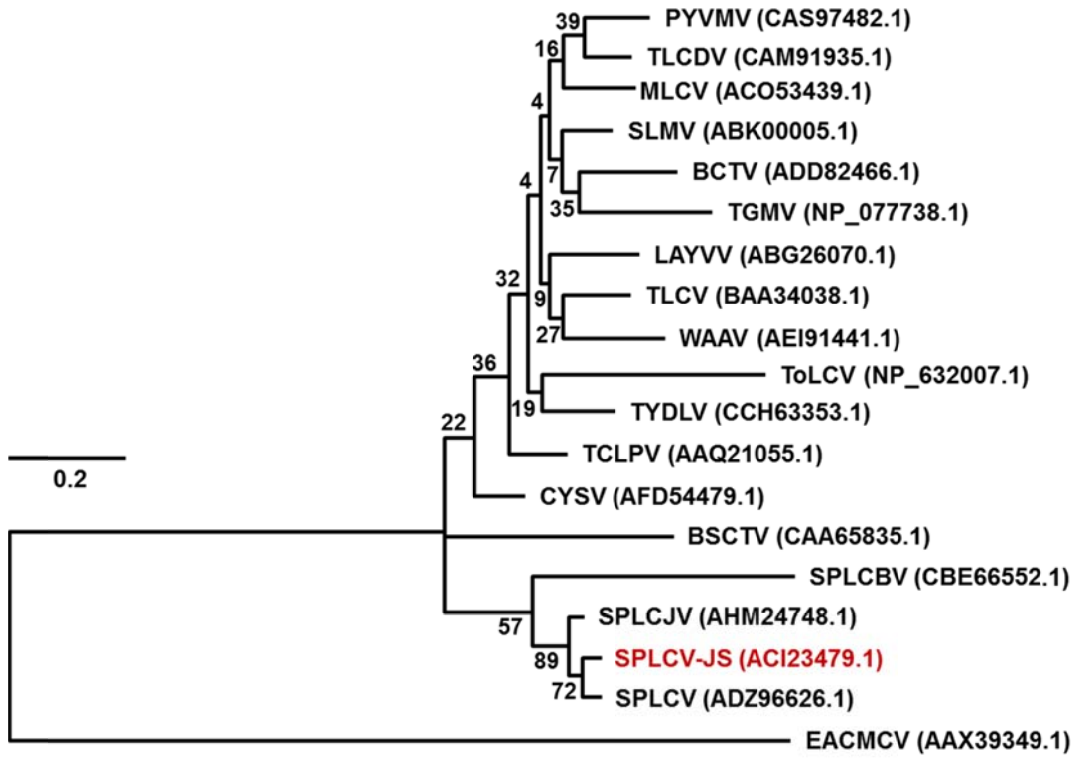

Supplementary Figure S1 Phylogenic tree of C4 proteins among major begomoviruses generated by the neighbor-joining method using MEGA 4.0.

Accession numbers for each C4/AC4 protein are indicated. The acronyms for the viruses used are as follows: PYVMV, *Pepper yellow vein Mali virus*; TLCDV, *Tomato leaf curl Diana virus*; MLCV, *Malvastrum leaf curl virus*; SLMV, *Soybean blistering mosaic virus;* BCTV, *Beet curly top virus*; TGMV, *Tomato golden mosaic virus*; LAYVV, *Lindernia anagallis yellow vein virus;* TLCV, *Tobacco leaf curl virus;* WAAV, *West African Asystasia virus 2;*ToLCV, *Tomato leaf curl virus;* TYDLV, *Tomato yellow distortion leaf virus;* TCLPV, *Tomato chino La Paz virus;* CYSV, *Centrosema yellow spot virus;* BSCTV, *Beet severe curly top virus*; SPLCBV, *Sweet potato leaf curl Bengal virus;* SPLCJV, *Sweet potato leaf curl Japan virus;* SPLCV-JS, *Sweet potato leaf curl virus*-Jiangsu*;* SPLCV, *Sweet potato leaf curl virus;* EACMCV, *East African cassava mosaic Cameroon virus*.

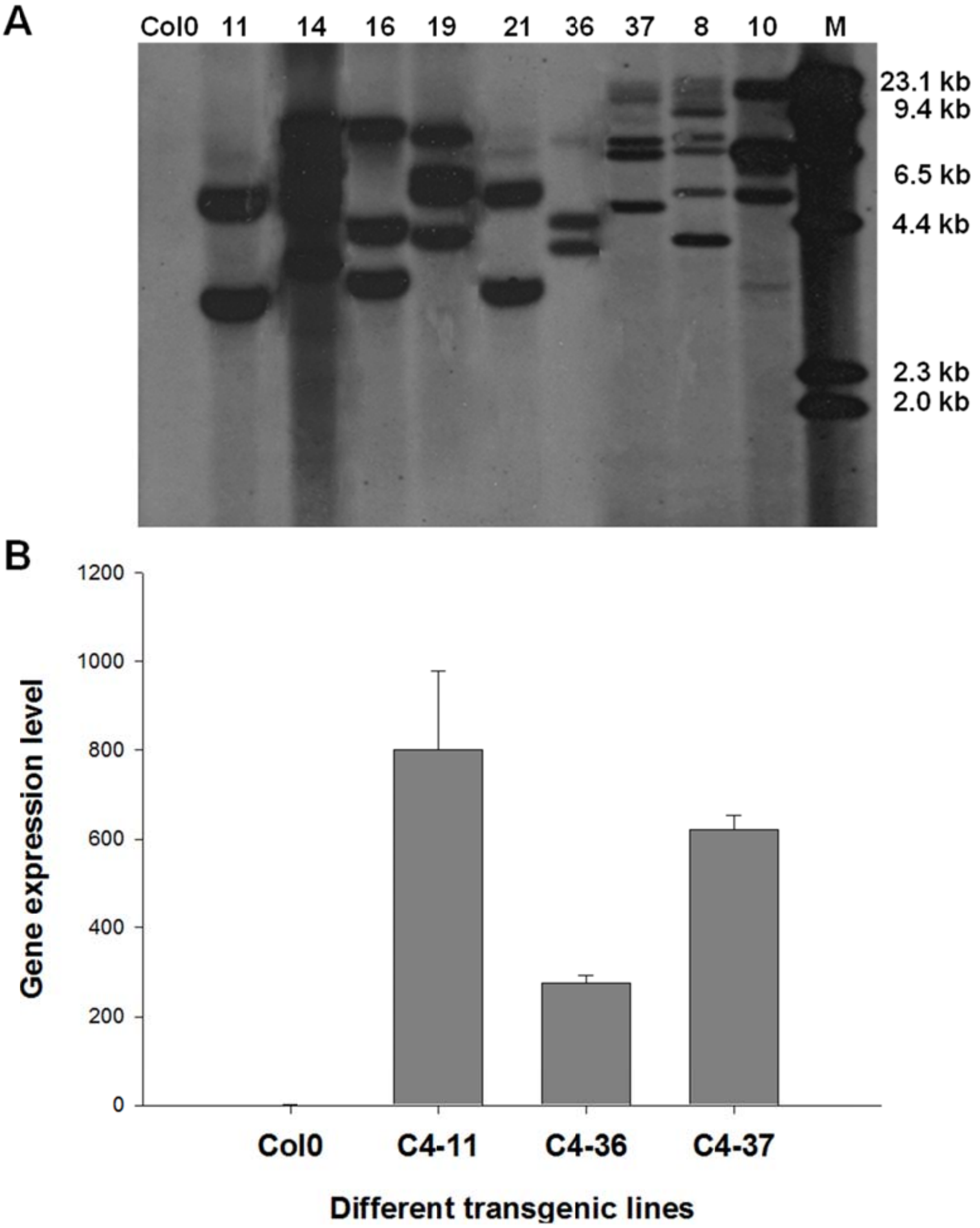

Supplementary Figure S2 Molecular analysis of SPLCV-JS *C4* transgenic plants.

(A) Southern blot analysis of the C4 transgenic lines. Genomic DNA was hybridized with a DIG-labeled probe specific for the C4 gene. M, λ- *Hin*dIII Marker, WT, Col0. 11, 14, 16, 19, 21, 36, 37, 8, and 10 indicate T1 transgenic lines. (B) Real-time qRT-PCR analysis of the expression level of C4 in C4-11, C4-36 and C4-37. Col0 was used as control. The *Arabidopsis* actin gene was used as an internal reference. Bars indicate the SE.

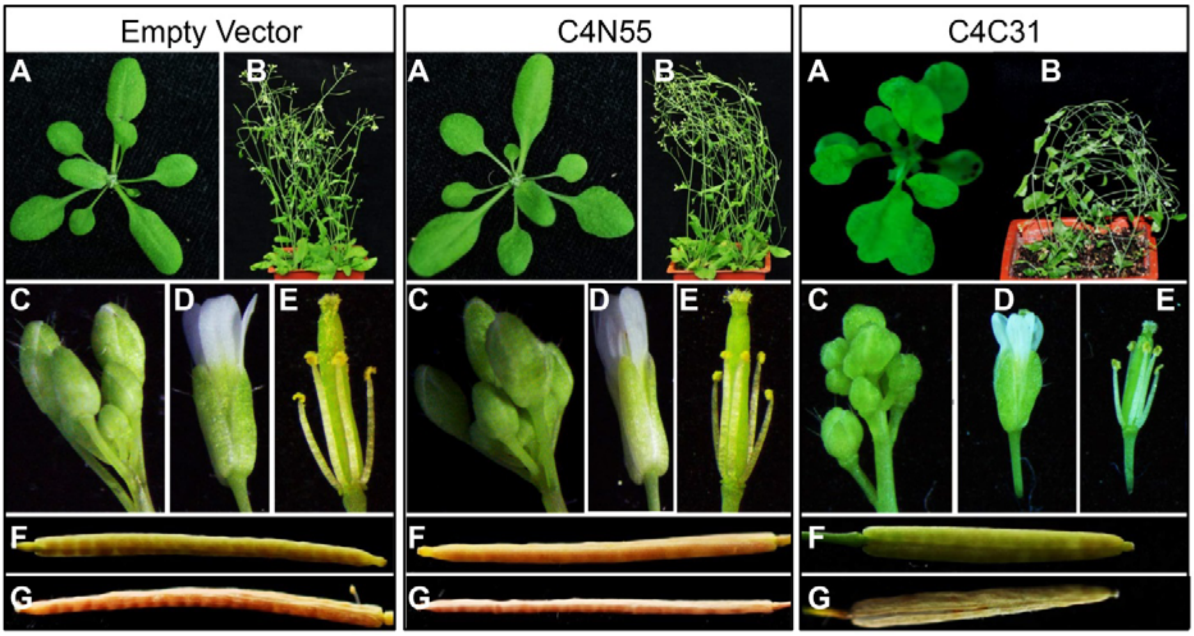

Supplementary Figure S3 Phenotype of transgenic *Arabidopsis* plant lines transformed by empty vector pCAMBIA1301, SPLCV-JS-C4N55 and SPLCV-JS-C4C31.

(A) Seedlings. (B) Flowering *Arabidopsis* plants. (C) Inflorescence. (D) Flowers. (E) Stamens. (F) Pistils. (G) Siliques.

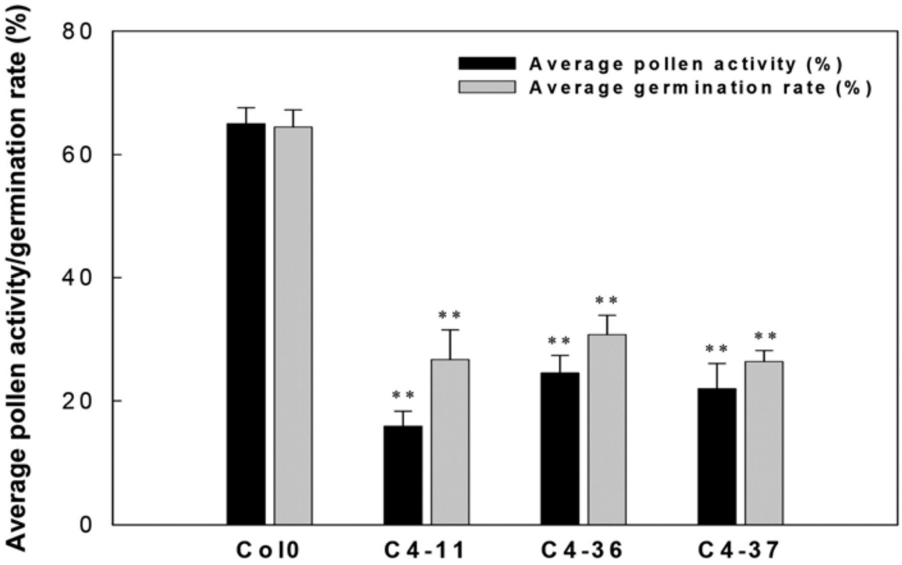

Supplementary Figure S4 Comparison of pollen activity and germination rate among SPLCV-JS *C4* transgenic *Arabidopsis* lines and the wild-type Col0.

Double asterisks indicate a statistically significant difference when compared with the wild type value according to the Student's *t* test (P<0.01).

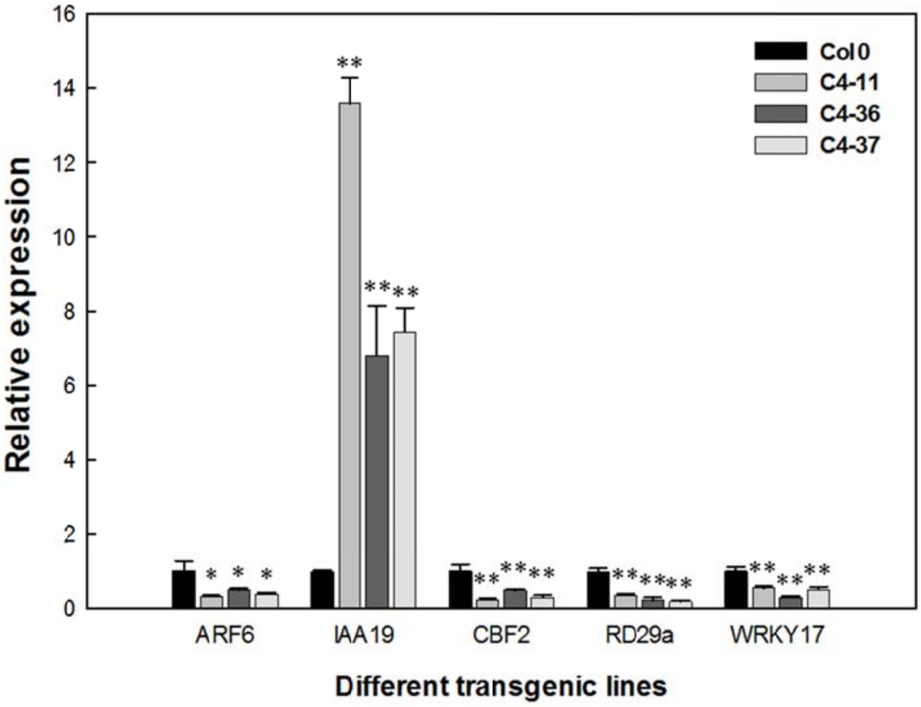

Supplementary Figure S5 Real-time qRT-PCR analysis of stress-related genes in the wild-type and SPLCV-JS *C4*-transgenic *Arabidopsis* lines.

The expression levels of *ARF6, IAA19, CBF2, RD29a* and *WRKY17* genes were detected and their expression level in Col0 was used as control. The U-BOX gene *At5g15400* was used as an internal reference. Data presented as mean ± SD. Asterisks indicate a statistically significant difference when compared with the wild type value according to the Student's *t* test (*, P<0.05; **, P<0.01).
